# Heterogeneity and Developmental Connections between Cell Types Inhabiting Teeth

**DOI:** 10.3389/fphys.2017.00376

**Published:** 2017-06-07

**Authors:** Jan Krivanek, Igor Adameyko, Kaj Fried

**Affiliations:** ^1^Department of Molecular Neurosciences, Center for Brain Research, Medical University ViennaVienna, Austria; ^2^Department of Physiology and Pharmacology, Karolinska InstitutetStockholm, Sweden; ^3^Department of Neuroscience, Karolinska InstitutetStockholm, Sweden

**Keywords:** odontogenesis, tooth, dental development, stem cells, cell heterogeneity, dental pulp, odontoblast, ameloblast

## Abstract

Every tissue is composed of multiple cell types that are developmentally, evolutionary and functionally integrated into the unit we call an organ. Teeth, our organs for biting and mastication, are complex and made of many different cell types connected or disconnected in terms of their ontogeny. In general, epithelial and mesenchymal compartments represent the major framework of tooth formation. Thus, they give rise to the two most important matrix–producing populations: ameloblasts generating enamel and odontoblasts producing dentin. However, the real picture is far from this quite simplified view. Diverse pulp cells, the immune system, the vascular system, the innervation and cells organizing the dental follicle all interact, and jointly participate in transforming lifeless matrix into a functional organ that can sense and protect itself. Here we outline the heterogeneity of cell types that inhabit the tooth, and also provide a life history of the major populations. The mouse model system has been indispensable not only for the studies of cell lineages and heterogeneity, but also for the investigation of dental stem cells and tooth patterning during development. Finally, we briefly discuss the evolutionary aspects of cell type diversity and dental tissue integration.

## Heterogeneity of the dental epithelial compartment

Epithelia are reasonably simple tissues, and yet they are very powerful. Controlled heterogeneity of the epithelial cell populations enables multiple variations of reasonably similar structures, and is necessary for the general composition of the tooth. Epithelium-based morphogenesis generates remarkable examples of complexity, found in e.g., feathers, hair follicles, multiple glands, and finally, teeth. Indeed, epithelia are dominant guides not only for themselves, but also for underlying cell types, for example dental mesenchyme. In teeth, multiple epithelial cell subtypes are interacting with other tissues, maintaining stem cell properties, producing the tissue bends, and generating ameloblasts—the key enamel-producing cell type.

Beginning at the very onset of tooth development, a rather uniform dental epithelium gives rise to a folded structure known as a dental lamina, which in turn yields a complex structure of quite different epithelial-derived cells. These include the cells of the inner (IEE) and the outer (OEE) enamel epithelium, which meet at the folded cervical loops. The IEE is a necessary interaction partner for underlying mesenchymal cells. Under the influence of IEE, the mesenchyme differentiates toward the odontoblast lineage, and conversely, the mesenchyme directs the IEE into ameloblast differentiation (Balic and Thesleff, 2015). Between the IEE and the OEE is a loosely composed cell assembly, the stellate reticulum, where stem cells that give rise to all mentioned epithelial compartments are located. The cells of the stellate reticulum are separated from the IEE by a very thin cell layer at the inner rim of the IEE, the stratum intermedium. The stem cells between the IEE and the OEE enable growth of the epithelium, and in continuously growing teeth they contribute to the regeneration of epithelium and stratum intermedium, respectively (Juuri et al., 2012).

Specification of the dental epithelium is initiated by mutual interactions with ectomesenchyme. The ectomesenchyme is derived from migrating cranial neural crest in places where teeth are going to develop (Koussoulakou et al., 2009). In this way, dental placodes arising from epithelial thickenings are formed. Further cell divisions of the dental epithelium give rise to tooth buds, which as yet consist of uniform cells (Koussoulakou et al., 2009). Differentiation and early shaping of the future tooth is initiated by the formation of the primary enamel knot, a signaling cell complex which arrests cell division in the most apical part of the bud, leading to proliferation in lateral parts only (Vaahtokari et al., 1996; Jernvall and Thesleff, 2000; Thesleff et al., 2001; Harada and Ohshima, 2004). With subsequent development, the tooth bud will advance into the cap stage, where the first differentiation of cells gives rise to the cervical loops with IEE/OEE as well as the stellate reticulum possessing stemness activity. Depending on future tooth type, other enamel knots are later formed which provide for the vast heterogeneity of all different tooth shapes that have evolved within different species (Jernvall and Thesleff, 2012).

In the continuously growing mouse incisor, the entire spectrum of developmental and adult stages of epithelial cells are seen throughout life. This includes SC's (stem cells) maintaining their niche and producing a highly mitotically active population of TAC's (transit amplifying cells). These non-differentiated cells are pushed into differentiation pathways by numerous molecular factors from the environment (mainly FGF, WNT, BMP, and Shh pathways), but also by influences from cell movements and physical forces in surrounding tissues. In spite of the fact that the initial phases of cell specification are more or less similar for both mesenchymal and epithelial compartments, the molecular mechanisms maintaining these niches are substantially different (Thesleff and Tummers, 2008; Koussoulakou et al., 2009).

**Figure 1:**
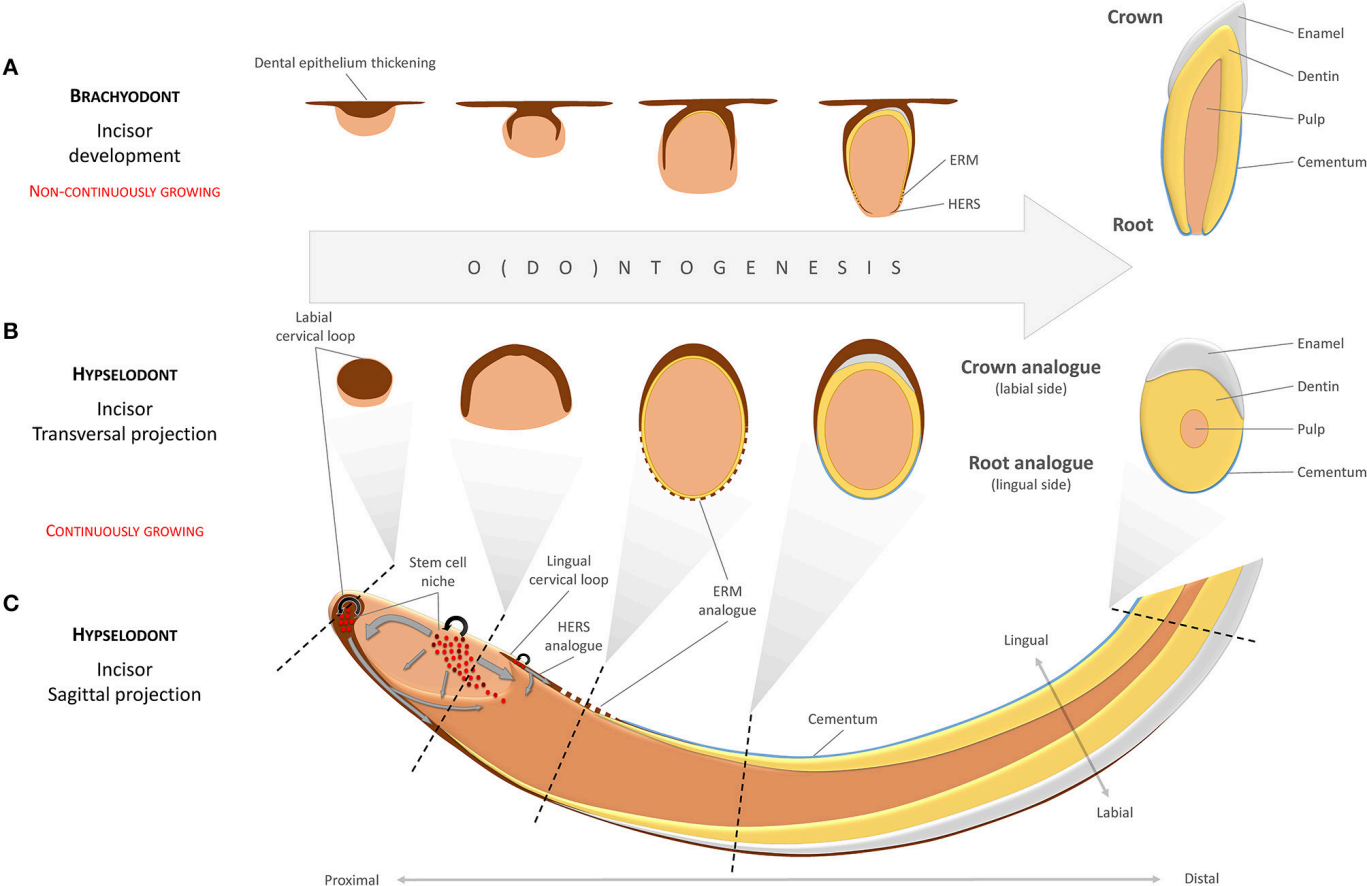
Developmental analogies between the continuously growing incisor and brachyodont tooth development. Different developmental stages in a brachyodont (non-continuously growing) tooth **(A)** are analogous to different transversal projections of a hypselodont (continuously growing) tooth along a proximal-distal axis **(B,C)**. The labial surface of the mouse continuously growing incisor, usually described as a “crown analog,” is covered by enamel. This is in contrast to the lingual side, the “root analog,” which is covered by cementum. Gray arrows indicate directions of movements in the mesenchymal and the epithelial cell populations. (ERM—epithelial cell rests of Malassez; HERS—Hertwig's epithelial root sheath).

Incisor-renewing epithelial stem cells are located in the most apical part of the tooth at the cervical loop, which consists of folded epithelium (the morphologically and functionally distinct OEE and IEE). The SC niche is lodged between the OEE and IEE, respectively (Harada et al., 1999; Juuri et al., 2012). There are obvious functional differences between the labial and the lingual surfaces of the incisor. In spite of the fact that both the labial (LaCL) and the lingual (LiCL) cervical loop possess the capacity to regenerate, only the LaCL is capable of forming functional enamel-producing ameloblasts on the side of the IEE (Figure 1). Consequently, since it generates enamel, the labial aspect is often called “crown analog.” In parallel to this, the LiCL, is referred to as “root analog” since it resembles developing roots that lack ameloblasts but possess cementoblasts and cementum (Stern, 1964; Warshawsky, 1968; Beertsen and Niehof, 1986; Balic and Thesleff, 2015). Using Sox2-traced mice, it has been shown that the slow-cycling stem cells located in the LaCL remain in their locus for long periods, and have the potential to continuously produce cells for both OEE, IEE, and functional ameloblasts (Juuri et al., 2012).

SC's are maintained through a highly complex process that depends on interactive signaling between dental papilla/pulp and epithelium. Distinct pathways and feedback loops are used for keeping SC's in the stellate reticulum active. FGF3 and FGF10 are major molecular factors in this process. They are produced by the mesenchyme near the cervical loop and subsequently interact with FGFR1b and FGFR2b receptors in the cervical loop (Harada et al., 1999, 2002, reviewed in Balic and Thesleff, 2015). Different knockout experiments have shown that LaCL renewal becomes impaired after targeting of FGF ligands or receptors (Wang et al., 2007; Klein et al., 2008; Lin et al., 2009). However, if negative FGF feedback loops controlled by Sprouty genes are disrupted in the pulp, enamel is produced also by LiCL (Klein et al., 2008). Other pathways that are important for maintaining the epithelial SC niche include Notch, BMP, and Wnt (reviewed by Tummers and Thesleff, 2003; Li et al., 2017).

During the past few years, a number of studies have appeared which have increased the understanding of the complex 3-dimensional aspects of the LaCL/LiCL and associated movements of cells that generate hard matrices (Charles et al., 2011; Juuri et al., 2012; Cox, 2013; Lesot et al., 2014). Sox2+ stem cells produce progeny that migrate to the enamel epithelium, but also cells that maintain the stellate reticulum itself. Cells are moving further in the labial-distal direction and differentiate into ameloblasts. This process is regulated by an Shh positive feedback loop. Cells are also migrating medio/latero-distally to the IEE/OEE ridge, a process which is marked by Wnt inhibitor SFRP5 expression (Seidel et al., 2010; Juuri et al., 2012). Populations of Sox2+, Sfrp5+, and Shh+ cells are subsequently located in different, non-overlapping positions (Seidel et al., 2010; Juuri et al., 2012).

**Figure 2:**
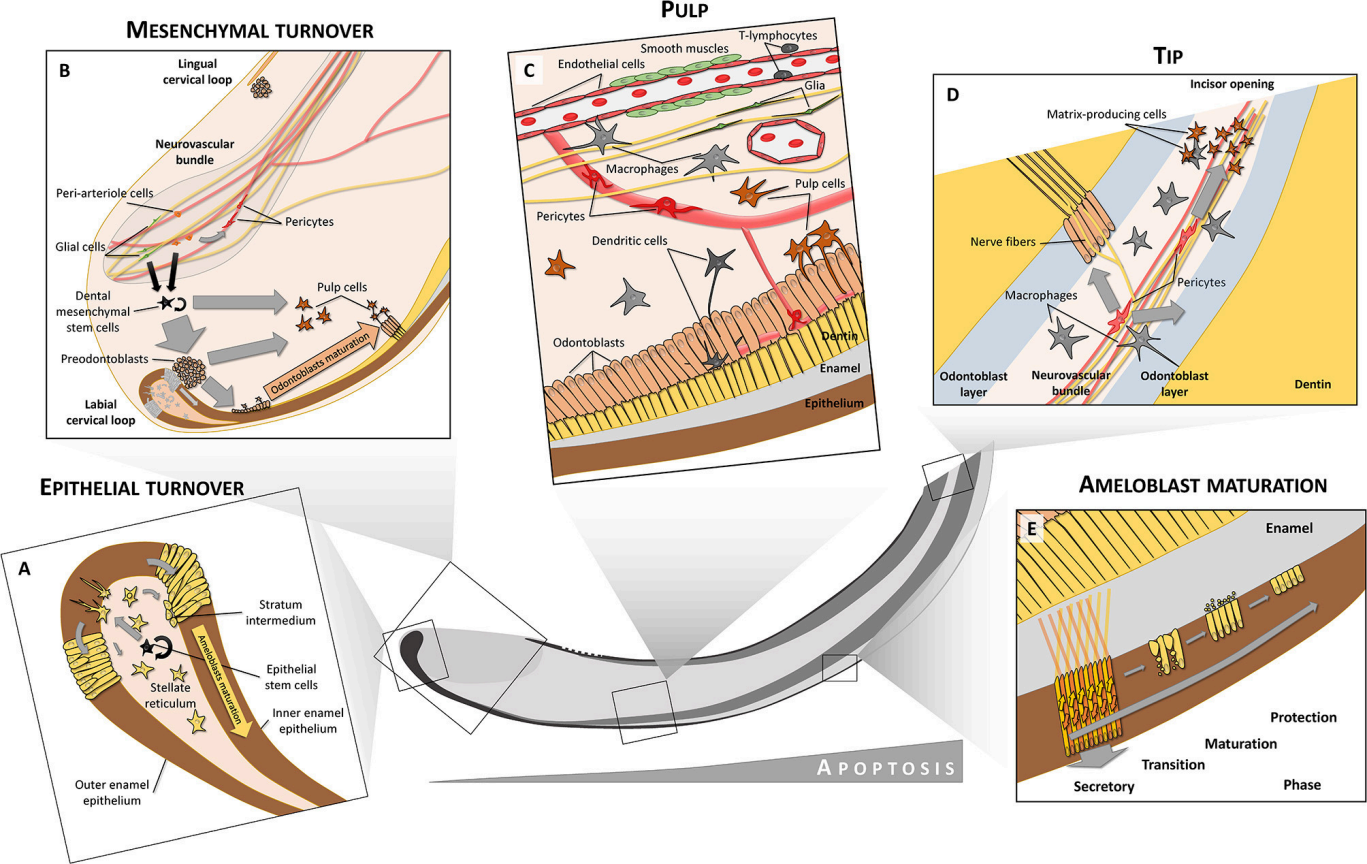
Cell heterogeneity in tooth. Both epithelium and mesenchyme preserve their stem cell niches **(A,B)**. In the labial cervical loop, stem cells are located in the stellate reticulum from where they produce transit-amplifying cells that are inserted into the dental epithelium and later differentiate into inner (IEE) and outer enamel epithelium (OEE). Only the labial IEE can give rise to fully functional enamel-producing ameloblasts. In contrast to dental epithelium, dental mesenchymal stem cells are derived from both glial cells and peri-arteriole cells located in a neurovascular bundle. Mesenchymal stem cells give rise to both odontoblasts and pulp cell progeny. Moreover, peri-arteriole cells can give rise to NG2^+^ pericytes. The pulp is formed and maintained by complex interactions between many different cell types, including circulatory system-related cells (endothelium, smooth muscles, pericytes, immune cells), nerves with axons and glia or odontoblasts, and related pulp cells **(C)**. Close to the tip of the incisor, increased apoptosis can be observed. Pericytes in the tip give rise to matrix-producing cells which help to seal the incisor opening **(D)**. A close structural relationship between odontoblasts and immune cells like macrophages and dendritic cells is seen in teeth **(C,D)**. After differentiation into secretory ameloblasts, when the full volume of enamel matrix is produced, these cells undergo a transition phase **(E)**. During this, about 25% of the ameloblasts die through apoptosis. The remaining ones become less elongated, and during the maturation phase they release inorganic compounds and mineralize enamel rods to full extent. After the maturation phase, only about 50% of the initial amount of ameloblasts remain viable to form a protective layer which finally disappears after tooth eruption. Ameloblasts during the secretory phase have a characteristic movement pattern (shown by arrows in **E**). Through combined movements outwards (pushed by matrix secretion) and toward the tip (pushed by newly differentiated ameloblasts) they form alternating layers where one layer slides between adjacent layers that move in opposite directions. This creates a characteristic criss-cross pattern (yellow and orange arrows in **E**).

The entire developmental history of ameloblasts (and also odontoblasts) is literally sealed in the inner structure of enamel (dentin respectively). Ameloblasts are moving during matrix secretion, but so far only little is known about the exact mechanism of this movement (Figure 2). Enamel is created by walled matrix blocks which form enamel rods during the ameloblast secretory stage. The rods become fully mineralized within the maturation phase (Smith, 1998; Zheng et al., 2014). The formation of enamel is characterized by a complex microstructure, making it the hardest tissue of the body. Hardness itself is dependent not only on a high content of hydroxyapatite crystals, but importantly, also on the micro-patterning of the enamel. Enamel decussation, where bundles of rods cross each other throughout the distance from the enamel-dentine junction to the outer enamel surface, is highly variable in different species. It is adapted to animal life conditions in order to provide specific hardness and ability to absorb mechanical energy. The extremely organized rod decussation in mouse enamel explains how it can be so hard in spite of its relative small size in comparison to e.g., human teeth. Human enamel, in turn, have different decussation patterns as compared to mouse enamel. There is a wavy structure in inner human enamel which is able to stop inward spreading of cracks from the straight rod structure of the outer enamel (Skobe and Stern, 1980; Bajaj and Arola, 2009). Interestingly, enamel protein secretion is under control of circadian “clock” genes which controls enamel production differently during day and night (reviewed in Zheng et al., 2014). During the entire secretory phase, each ameloblast produces one enamel rod to form organized matrix with desired mechanical abilities. When the secretory stage is over, one quarter of ameloblasts undergo apoptosis and after the maturation phase only half of the ameloblasts remain to form a protective layer, which disappears completely in human teeth during eruption (Smith, 1998). Some evidence suggests that TGF-β1 with the Smad2/3 signaling pathway are involved in the induction of apoptosis in the maturation phase (Tsuchiya et al., 2009).

Thus, the heterogeneity and functional division between epithelial subtypes in the embryonic and mature teeth provides for tissue-tissue interaction and production of hard matrix at the correct time and location. It enables shaping and morphogenesis, self-renewal, growth and many other functions. Generation of epithelial subtypes in the tooth is not well understood and requires extensive further investigations.

**Key papers:**

- Balic and Thesleff (2015).
- Cox (2013).
- Yu et al. (2015).
- Koussoulakou et al. (2009).

## Heterogeneity of the mesenchymal compartment

The mesenchymal compartment of the fully formed tooth includes various cell types and subtypes that are largely represented by odontoblasts, diverse pulp cells and dental mesenchymal stem cells together with transiently amplifying cells (if retained). Hence, the pulpal cells have multifarious identities, originating in an intricate developmental history.

Mouse tooth development is based on dynamic interactions between epithelial and mesenchymal compartments. The mesenchymal compartment consists of the condensed mesenchyme during early embryonic development. This mesenchyme will eventually differentiate into pulp tissue and dentin-producing odontoblasts.

Mouse molars are stationary during adult life in terms of cell dynamics, while the incisors keep growing throughout the entire life. During this growth, pulp cells and odontoblasts are renewed by dental mesenchymal stem cells that reside in the area between the cervical loops at the tooth apex. Recently it was discovered that these mesenchymal stem cells are heterogeneous and demonstrate differences in terms of markers that are expressed and in the amount of progeny. For instance, slow cycling cells in the dental pulp of mouse incisor express Thy1 (CD90). In line with this, genetic tracing with Thy1-Cre has demonstrated a long-term presence of the Thy1+ progeny in the tooth (Kaukua et al., 2014). This progeny consisted of 10–20% of all odontoblasts and pulp cells, corresponding to the actual proportion of Thy1+/slow cycling cells among all slow cycling cells at the tooth apex (Sharpe, 2016).

Another marker that co-localizes with slow cycling cells is Gli1 (Zhao et al., 2014). In line with the results obtained with Thy1+ cells, lineage tracing in the Gli1-CreERT2 mouse line demonstrated the presence of a long-lasting population consisting of pulp cells and odontoblasts. The proportion of Gli1-traced cells appeared to be very high, close to 100%. Dental mesenchymal stem cells expressing Gli1 are located around and inside the neuro-vascular bundle, and are Shh-dependent. One of the key sources of Shh inside of the tooth has turned out to be pulpal sensory nerves (Zhao et al., 2014). Accordingly, experimental denervations of mouse incisors result in tooth growth arrest, confirming that the nerve is important for tooth growth and self-renewal (Kaukua et al., 2014; Zhao et al., 2014).

The actual developmental origin of dental mesenchymal stem cells is still unclear. It is obvious that they are progenies of the neural crest. However, it is not yet established whether most of the dental mesenchymal stem cells form at the end of embryonic development, when general proliferation decreases inside of the tooth, or if these stem cells segregate much earlier and then carry out their key role postnatally. Some of the dental mesenchymal stem cells are supplied by the innervation (see below). Peripheral glial cells can be recruited into the mesenchymal stem cell niche postnatally in low numbers. During early age the contribution of these glia-derived mesenchymal cells to odontoblasts and pulp cells is relatively low, but it tends to increase toward the end of the animal's life (Kaukua et al., 2014).

Much attention has been focused on the identification of dental mesenchymal stem cells *in vitro* and *in vivo* after damage. However, these studies often do not relate directly to the physiological *in vivo* tooth self-renewal situation (Sloan and Waddington, 2009). At present, it seems that further long-term lineage tracing experiments are needed in order to resolve this issue.

Clonal genetic tracing experiments involving color multiplexing with Confetti reporters demonstrated that an individual mesenchymal stem cell is bipotent, and can give rise to both pulp and odontoblast fates. Recent data suggests that this fate selection depends on the extrinsic signals potentially provided by the epithelial compartment. Thus, odontoblasts are induced only in association with the epithelial layer at the tooth apex (Kaukua et al., 2014). Further studies of the regulation of the apical stem cell compartment that produces spatially defined population of transiently amplifying progenitors will hopefully elucidate at which level of cellular hierarchy the fate split occurs.

Odontoblasts undergo further maturation and reorganize their branched processes simultaneously with intense matrix production. In the mature phase, odontoblast express certain ion channels and other markers, which suggest that they may subserve a sensory function (reviewed in Chung et al., 2013). This could be achieved through communications with associated nerve fibers and/or through interactions with immune cells.

Mature odontoblasts from mouse incisors demonstrate heterogeneity in terms of cell configuration: a fraction of odontoblasts appear pyramidal in shape with their nuclei in a position next to the matrix and without any process entering into the dentinal tubule (Khatibi Shahidi et al., 2015). The heterogeneity of other mesenchymal cells in the mature dental pulp is not well understood. Among those with a hitherto unknown identity are perivascular pulp cells that contact pericytes, and morphologically aberrant cells in the layer immediately below the odontoblasts. These latter cells project fine processes deep into the odontoblast layer toward the hard matrix (Khatibi Shahidi et al., 2015). The function of these projections is unclear.

Thus, the heterogeneity of the mesenchymal compartment is much higher than is commonly thought, starting from different subtypes of stem cells and extending all the way to morphologically diverse populations of odontoblasts.

**Key papers:**

- Sharpe (2016).
- Sloan and Waddington (2009).

## Cell types of the dental follicle and root formation

The root system anchors the tooth to the alveolar bone of the maxilla or mandible. It transfers occlusal forces to the jaw bones, and monitors these forces through an elaborate periodontal proprioceptive innervation (Trulsson and Johansson, 2002). The cells that give rise to root tissue are of both epithelial and mesenchymal origin, but the epithelium has mainly signaling functions. The mesenchymal cells differentiate along distinctly dissimilar paths and form pulp, dentin, cementum and the periodontal ligament. The diversity and putative varying functions among the cell types that create these different tissues are largely unknown. Likewise, it is not known in detail how they differ from similar cell types in other locations, e.g. cementoblasts vs. odontoblasts or osteocytes.

During early odontogenesis, cells at the periphery of the condensed dental mesenchyme form the dental follicle. In teeth that do not grow continuously, these cells will differentiate into cementoblasts and periodontium and produce the root segments of the tooth. In this process, the cervical loop will lose its central cellular content so that only a double layer of basal epithelium remains (the epithelial diaphragm). This double layer constitutes Hertwig's epithelial root sheet (HERS), an important structure in root development, responsible for shaping and scaling of roots by physical division of the dental papilla and the dental follicle (Xiong et al., 2013). After matrix production by odontoblasts has been commenced, HERS is fenestrated into small fragments and remains in the periodontal connective tissue as the epithelial cell rests of Malassez (ERM) (Figure 1). The ERM seems to plays an important role in periodontal ligament homeostasis, and contributes to alveolar bone remodeling (Diekwisch, 2001; Luan et al., 2006).

Neither HERS nor ERM seem to have much potential for further growth, but HERS plays an important role in root elongation by secreting Shh. This secretion, which is under the control of BMP/TGFbeta/SMAD signaling, probably safeguards appropriate levels of Shh in the dental mesenchyme that forms the root (Nakatomi et al., 2006; Huang et al., 2010). Thus, experimental manipulations of Shh in this region results in shortening of the root (Liu et al., 2015). Furthermore, Wnt/beta-catenin and BMP/TGFbeta activity in odontoblasts and dental mesenchyme influence root growth, although the precise mechanisms involved are still unclear (Kim et al., 2013; Rakian et al., 2013; Wang et al., 2013).

The properties of the mesenchymal progenitors from the dental follicle that eventually will give rise to the tissues of the root have not been fully understood. There seem to be clearcut similarities between osteoblast and cementoblast differentiation, since both Runx2 and Osterix (Osx) have vital functions in these two processes (Nakashima et al., 2002). Conditional deletion of Osx in dental mesenchymal cells leads to a reduced cellular cementum formation and lowered dentin matrix protein (DMP1) gene expression in cementocytes (Cao et al., 2012).

Recently, in a series of lineage tracing and genetic labeling/mutation studies, it was shown that Osx-expressing mesenchymal dental follicle progenitors contribute to all root tissues: odontoblasts, pulp cells, cementoblasts and some periodontal ligament (PDL) cells (Ono et al., 2016). Furthermore, cementoblast differentiation and tooth root formation requires the parathyroid hormone/parathyroid hormone-related protein (PTHrP) receptor PPR in these progenitors. PTHrP is expressed in the dental follicle as well, and it was suggested that its primary target is Osx-lineage progenitors involved in PPR signaling. PTHrP can in this way influence root formation, tooth eruption and PDL attachment (Ono et al., 2016), which is underlined by the fact that an autosomal dominant mutation of the PPR causes a primary failure of tooth eruption in human (Frazier-Bowers et al., 2014). However, detailed information concerning the time-related activities of the PTHrP-PPR signaling system in cementogenesis, general root formation and tooth eruption is still lacking.

Membrane-type matrix metalloproteinase 1 (MT1-MMP) is also indispensable in the organization of the dental follicle/PDL region and dental root development. Thus, a selective knock-out of MT1-MMP in Osx-expressing mesenchymal cells yielded multiple defects: short roots, defective dentin formation and mineralization, and reduced alveolar bone formation. This suggests that MT1-MMP activity in the dental mesenchyme is vital for tooth root formation and eruption, e.g. by processing signaling molecules that affect Wnt and Notch signaling pathways during dental development (Xu et al., 2016).

To summarize, spatiotemporal interactions between epithelium and mesenchyme will in due course form the dental follicle and subsequently advance the establishment of root dentin, cementum and periodontal tissues. Complex molecular networks that involve a multiplicity of cell types will determine features necessary for adequate function at specific sites, such as root number, shape and trajectory.

**Key papers:**

- Diekwisch (2001).
- Huang et al. (2009).
- Li et al. (2017).

## Cell types related to innervation and vascular system

Teeth are complex organs that require a vascular system to transport gases, nutrients and metabolites as well as a sensory system to, among other things, control biting strength. Indeed, the abundant pulpal innervation is a hallmark of the mammalian tooth. This is perhaps not surprising, if one considers the proposal that the tooth is a modified pre-historic primary mechanosensory organ (Northcutt and Gans, 1983). Throughout millions of years, its pulpal nerve fibers may gradually have evolved into transducers of signals that evoke pain sensations only (see Fried et al., 2011).

The innervation of the mouse tooth provides a very useful prototype system for studies of mammalian nerve-target interactions during development, in adult life homeostasis, after injury and in senescence. The mouse possesses two types of teeth with different innervation patterns. The three molars develop, function and are innervated much as other mammalian molars, including human. The continuously growing rodent incisor renews its tissue on a monthly basis, thus offering a model structure that includes highly active stem cell regions throughout adulthood. Its innervation can best be described as permanently immature.

During fetal life, pioneer trigeminal axons are present in the branchial/pharyngeal arches at the earliest stages of mouse development, well before the initial signs of tooth development are evident. These axons branch and innervate the developing jaw processes, but avoid the areas of mesenchymal condensations that mark the sites of tooth formation (reviewed by Fried and Gibbs, 2014). The issue of whether nerve fibers actually may have an inductive role in tooth initiation, as is the case in e.g. salivary glands (Nedvetsky et al., 2014) is still unresolved. Tooth explants that are removed grow without nerve fibers *in vitro* (Lumsden and Buchanan, 1986; Sun et al., 2014), but it cannot be excluded that nerves influence the ectomesenchyme or dental epithelium earlier, when teeth actually are induced. To experimentally address this in the mouse, however, would require sophisticated nerve ablation experiments that as yet have not been performed.

Throughout fetal development, mandibular and maxillary sensory innervation proceeds. Basket-like axonal arborizations form around the incisor and molar tooth buds. However, although surrounding submucosa and other soft tissues become highly innervated, no axons enter the condensed dental mesenchyme/dental papilla until several days postnatally. This is in spite of the fact that the future dental pulp is among the most densely innervated tissue in the body. Unexpectedly and perhaps explaining the delay in tooth sensory innervation, these nerve networks have been shown to serve as stem cell niches for the developing tooth.

Textbooks have for decades taught that dental mesenchyme and odontoblasts are derived directly from migrating cranial neural crest cells. However, recent data have established an additional important glial source. Using genetic tools in mouse model systems, it was shown that multipotent Schwann Cell Precursors (SCP) of the axonal networks that are associated with the tooth anlagen leave their nerve branches and contribute with pulp cells as well as odontoblasts in clonal configurations.

For experiments, advantage was taken of the fact that PLP1 and Sox10 are expressed in cranial neural crest, but after migration around embryonic days (E)9–10, they are retained in SCPs and not in mesenchyme. Lineage tracing studies with PLP-CreERT2 and Sox10-CreERT2 mice where recombination was induced at E12.5 and/or later showed traced SCP in peripheral nerves close to tooth placodes. Around early and late bell stage, traced SCP-derived progeny formed streams of cells consisting of dental mesenchymal stem cells, pulp cells and odontoblasts in the developing tooth. A similar mechanisms whereby trigeminal nerve glia contributes with pulpal cells continues to operate during adult renewal of the continuously growing mouse incisor (Kaukua et al., 2014).

A complex interplay between deployed incisor pulpal stem cells and nerves is indicated by the fact that Shh secreted from nerve endings within the neuro-vascular bundle seems to warrant continuous tooth growth. This is probably by acting on associated Gli1^+^ dental mesenchymal stem cells (including SCP), see Figure 2B (Zhao et al., 2014). Presumably, such a mechanism is at hand during embryonic odontogenesis as well.

In contrast to nerve fibers, blood vessels, as detected by endothelial markers, are present in mouse molars as early as at E16 (early bell stage; Nait Lechguer et al., 2008). These blood vessels are composed of mesoderm-derived endothelial cells, which invade the dental papilla at the late cap stage, and form the vascular network *in situ*. Thus, the blood supply is created through vasculogenesis (newly formed vessels) rather than angiogenesis (ingrowth of vessels from surrounding pre-existing vessels; Rothová et al., 2011). It is unclear whether these early blood vessels serve as guiding cues for the axons that arrive later. However, it seems likely that true pulpal neurovascular bundles that carry arteries form only later. Thus, in limb skin, the pattern of peripheral nerve branching provides a template for branching of the emerging arterial vascular network. The specification is performed through a loop between nerve-derived VEGF and the endothelial VEGF-coreceptor NRP1 (Mukouyama et al., 2005). The same process may occur in the dental papilla/pulp once axons have entered.

In adult tissue, dental pericytes have attracted specific interest as a potential source of mesenchymal stem cells. They dwell on the abluminal surface of endothelial cells in the microvasculature of the pulpal tissue. These cells in mouse incisor have the capacity to transform into odontoblasts in response to injury, but also it has been recently shown that in non-continuously growing molars perivascular αSMA^+^ cells are able to differentiate into odontoblast to seal the injury site (Feng et al., 2011; Vidovic et al., 2017). Mouse incisors are continuously abraded during the lifespan, and undergo constant damage at the tip leaving exposed pulp underneath. It was recently shown that the opening of the tip of the incisor is continuously sealed with dentin-like matrix which is produced by cells that differentiate from perivascular pericytes (Pang et al., 2016). NG2-CreERT2 and Nestin-Cre genetic tracing showed that large numbers of pericyte-derived cells are produced within the tip, and some of them have odontoblast-like shapes. Thus, pericytes at this site serve as a reservoir for reparative cells, and help to maintain tissue homeostasis.

The spatial and temporal patterns of dental papilla pericyte development is not known, and consequently it is not known if pericytes have any specific roles in the organogenesis (aside from vasculogenesis) of the tooth.

Taken together, in addition to a number of classical functions performed by the vascular and nervous systems, the heterogeneity of nerves, glial cells and vessel-associated cell types accommodates for advanced reparative capacities and self-renewal of teeth.

**Key papers:**

- Kaukua et al. (2014).
- Pang et al. (2016).
- Zhao et al. (2014).
- Vidovic et al. (2017).

## Diversity of immune cells dwelling in the pulp

As mentioned above, the dental pulp is far from being a homogeneous tissue. Although most of the pulp is composed of mildly diverse cells of the mesenchymal compartment, it also hosts the immune system which has an as yet uncertain degree of cellular heterogeneity. The fact that the pulp is encased in a mineralized cavity makes it demarcated and suitable for both general and pulp-specific studies of the immune system during development, self-renewal, infection and homeostasis.

The environment within the tooth must be accurately surveyed for signs of infectious invasion. Knowledge of the immune cell signature of the pulp is vital in efforts to find new ways to protect teeth from infections. Additionally, immune cells may potentially influence hard matrix repair. The innate immunity response to bacteria invading the teeth includes the arrival of phagocytic cells and the generation of various inflammatory cytokines. When a dental infection becomes chronic, the adaptive immunity response kicks in: different subtypes of T-cells infiltrate the inflamed teeth (AlShwaimi et al., 2009). The most abundant T-cell type in the dental pulp is CD8+ cells (Hahn et al., 1989; Izumi et al., 1995; Sakurai et al., 1999). Their functions in non-inflamed tooth are currently unclear, although it is known that these cells are potent protectors against viral infections and are capable of augmenting phagocytosis. CD8+ T-lymphocytes are migratory, and can travel long distances from the site where they initially encountered an antigen (Marshall et al., 2001; Masopust et al., 2004). Some studies suggest that their main function in the pulp is immunosurveillance (extensively reviewed in Hahn and Liewehr, 2007).

Treg lymphocytes, a population known to prevent autoimmunity, are also found in low numbers around the roots of mouse molar teeth. However, in the case of tooth damage with periapical lesions, Foxp3-expressing cells (Foxp3 is a master gene for Treg) infiltrate the region around the lesion and dramatically increase in numbers in a time-dependent fashion. Additionally, these cells appear in large quantities in cervical lymph nodes after initiation of tooth pulp infection. This strongly suggests that Treg lymphocytes might play some role in the local immune response and the general regenerative capacity in teeth (AlShwaimi et al., 2009).

Macrophages and dendritic cells together represent one of the major immunocompetent cell types that are protective against dental infection and are necessary to remove senescent cells. These phagocytic cells are widely spread throughout the pulp cavity and can also project into dentinal tubules. During late embryonic development, around embryonic day 16, F4/80^+^ and CD68^+^ macrophages invade mouse molars and then continue to increase their numbers. M-CSF secreted by pulp cells plays a significant role in maintenance and proliferation of resident macrophages. It has been shown that a majority of the mouse pulpal macrophages proliferate locally and maintain their pool without additional recruitment from the blood supply (Iwasaki et al., 2011). Furthermore, secretion of OPN (Osteopontin) and M-CSF by immune cells correlates with the deployment of macrophages along the pulp-dentin border exactly when reparative matrix allocation commences in a mouse model of tooth transplantation. It was suggested that secretion of these soluble ligands that influence macrophages can stimulate differentiation of odontoblasts and production of reparative dentin (Saito et al., 2011). Other studies also highlighted the possibility that macrophages and dendritic cells might regulate the function and differentiation of odontoblasts (Ohshima et al., 1995; Tsuruga et al., 1999; Nakakura-Ohshima et al., 2003).

Dendritic cells demonstrate an exceptional capacity to capture antigens with their long filaments, and to process them. Two types of dendritic cells seem to be present in the dental pulp: CD11c^+^sentinel and F4/80^+^ interstitial cells (Zhang et al., 2006). The CD11c+ dendritic cells express toll-like receptors two and four, they are highly migratory and can move around the pulp in case of infection or even travel to the lymph nodes. Quite on the opposite, F4/80+ cells show low migratory activity and are rather residential. Also, these subtypes of immune cells show regional differences in their distribution inside of the mouse molar tooth (Zhang et al., 2006). Most of the pulpal dendritic cells are coagulation factor 13a (FXIIIa)+ (Nestle et al., 1993; Valladeau and Saeland, 2005) and can be further subdivided into a larger CD14+/CD68+ group and a smaller CD14+/CD68+/CD1a+ subpopulation (Okiji et al., 1997). Some HLA-DR+ dendritic cells appear negative for FXIIIa/CD1a markers (Hahn and Liewehr, 2007). Dendritic cells are often identified at interfaces, including the border between pulp and odontoblast layer as well as along large blood vessels. Dendritic cells spend only a part of their time inside of the dental pulp. After local surveillance activity and detection of e.g., bacterial antigens, they travel to the lymph nodes via lymphatic vessels and present the antigens to T-lymphocytes (Okiji et al., 1997; Randolph et al., 2002; Hahn and Liewehr, 2007; Bhingare et al., 2014).

When a tooth loses its nerve supply, the numbers of immune cells drop. This raises various questions regarding interactions between local nerves and the immune system (Fristad et al., 1995). In line with this, dendritic cells increase in numbers as the density of dental nerves increases during caries progression (Sakurai et al., 1999).

In general, very little is known about when and how various immune cell populations arrive to the mouse teeth during development. For many of these dental immune cell types we still do not know their immediate and precise developmental origin although all of them are generated during late embryonic and postnatal haematopoiesis. Finally, it is not clear if there is any substantial difference in immune cell type composition between continuously growing incisors and molars. These issues require further investigations.

**Key papers:**

- Hahn and Liewehr (2007).
- Farges et al. (2015).
- Perdiguero and Geissmann (2016).

## Evolutionary aspects of cell type heterogeneity in teeth

Every tissue or organ has its evolutionary origin. The composition of a tissue, in terms of cell types, stands behind the overall functionality. Heterogeneity of cell types increases throughout the evolution in every functional entity. Novel cellular functions are elaborated, while older functions become more and more discretely partitioned to the specializing cells during increasing labor division as a function of evolutionary time (Arendt et al., 2016).

The key events of the origin of teeth are the elaboration of dentin (odontoblast) and enamel (ameloblast), and the development of epithelial-mesenchymal interactions that enable the construction of the morphogenetically complex entities.

Apparently, when it comes to the competence of the epithelium to engage mesenchyme into complex morphogenesis, teeth are not unique among the different epithelial appendages (Hughes et al., 2011). Indeed, various skin glands, hairs and feathers demonstrate a very conserved signaling and initiation mechanisms including involvement of the EDAR pathway or expression of Shh at the early steps (Pispa and Thesleff, 2003; Mikkola, 2011). Such an archetypical developmental program shared by several distinct appendages suggests their common origin from some prototypical gland-like structure. In line with this, differences in tooth histology and geometrical features between different species represent the outcomes from minor tinkering with signaling pathways rather than being derived from major genetic divergences (Tummers and Thesleff, 2009). A conserved pattern is seen not only in the morphological and/or molecular similarities between groups that have functional teeth (mammals, reptiles, amphibians or fishes) but also in recent birds despite the fact that they lost the ability to form teeth some 70–80 million years ago. Still, birds retain the evolutionary conserved tooth-forming pathways in both oral epithelium and ectomesenchyme. *In vitro* co-culture and *in vivo* transplantation experiments demonstrated that chick epithelium can initiate dental development when combined with mouse mesenchyme and vice versa (Wang et al., 1998; Mitsiadis et al., 2003). While the origin of epithelial-mesenchymal interaction can be logically envisioned and even experimentally tested in the future, the path leading to the emergence of fundamental cell types, such as odontoblasts or ameloblasts, is neither simple nor intuitive.

In light of this, the evolution of odontodes and the evolution of neural crest fates represent two important lines deserving a brief discussion.

Two different theories exist regarding the origin of tooth-forming epithelium (Soukup et al., 2008; Fraser et al., 2010). Historically, teeth were first considered to be some kind of external odontodes originating from the ectodermal epithelium. This “outside-in” theory was based on the structural similarity between teeth and odontodes covering the bodies of jawless fishes. Indeed, odontodes often show analogous structural characteristics, including dentin that originates from ectomesenchyme and enameloid that closely resembles enamel of extant vertebrates. An opposing “inside-out” theory suggested that teeth originated from endodermal epithelium. It was based on findings that teeth-like structures were present deep in the pharynx even in the ancient jawless fishes (Soukup et al., 2008; Koussoulakou et al., 2009). By now, it seems rather likely that dentin and enamel as matrix types were elaborated during the early evolution of odontodes. This is supported by the example of *Psarolepis romeri*, an early Devonian bony fish, that had enamel-covered dermal odontodes and teeth covered with only dentin. The presence of enamel covering dentin as well as the similar situation with ganoin covering odontode scales suggest that such a composite structure preceded the combination of enamel and dentin in primitive teeth. *Lepisosteus oculatus* (the spotted gar) shows an epidermis-localized expression of enamel-specific genes. These genes are likely used to build ganoin—a matrix similar to enamel (Qu et al., 2015). In line with this, in another ancient stem osteichtian, *Andreolepis hedei*, the odontodes demonstrate highly regular growth mechanisms similar to the actual dentition in gnathostomes (Qu et al., 2013). However, it does not clarify whether the odontoblast preceded the osteocyte as a cell type or if it was elaborated with the help of bone-mineralizing genetic programs co-opted by osteocytes.

Developmentally, odontoblasts, similar to osteocytes and dermal fibroblasts, originate from migratory multipotent cranial neural crest cells. At the same time, current consensus suggests that neural crest cells incrementally acquired different fates throughout evolution (Hall, 2013). Therefore, the emergence of odontoblasts could occur based on the prototypical innervated neural crest-derived sensory cells (Baker et al., 2008; Magloire et al., 2009) surrounded by rather chaotic mineralized matrix produced by them or other bone-specific cell types in the external armor. As for the origin of ameloblasts, the primary function of epidermal units could be the patterning of underlying mesenchyme and induction of only dentin-based odontodes (quite spread in extinct fishes). Such patterning of placode-forming epithelium could invent the unique enamel-forming gene expression program (Qu et al., 2015) and in addition to its signaling role start to produce the enamel or ganoin-based covering of dermal odontodes. The more ancient history and the exact origin of the enamel-forming gene expression program should be studied further to deepen this area of investigation.

Over time, biting and chewing takes its toll, and teeth will inevitably deteriorate due to attrition, trauma and other insults. In order to compensate for this, different evolutionary strategies have emerged and accommodated for a gradual renewal of functional teeth. One way is to develop one or more novel generation/s of teeth. Based on the number of newly produced groups of teeth, species can be divided into mono-, di- and polyphyodonts. Some species have only one generation of teeth—*dentes decidui*—which is sufficient for the entire lifespan. Certain bats, shrews, seals and murid rodents belong to this group (van Nievelt and Smith, 2005; Jernvall and Thesleff, 2012). Humans, like most other mammals, have two generations of teeth. The first, *dentes decidui*, are later replaced by *dentes permanentes*. This strategy can be pushed to the extreme in case of polyphyodonts maintaining a postembryonic dental lamina capable of a continuous teeth renewal. This is in contrast to monophyodonts and diphyodonts, where the dental lamina is degraded after initiation of the primary and the secondary dentition, respectively (Buchtová et al., 2012; Gaete and Tucker, 2013). This type of tooth renewal is widespread among various vertebrates including reptiles, amphibians and fishes. *A. hedei*, the extinct stem osteichtian, shows a very similar archetypical tooth shedding dynamics, which confirms the ancient nature of this mechanism (Chen et al., 2016).

An alternative way was provided by the emergence of the continuously growing (hypselodont) tooth. These teeth are capable to maintain their stem cell niches and can grow throughout the whole lifespan of the animal. Such growing teeth are found in different species of rodents, lagomorphs, or tusk-bearing animals like elephants, boars, warthogs, walruses and even narwhals. This strategy would require a significant shift in heterogeneity of adult teeth, and the formation of epithelial as well as mesenchymal stem cell niches with associated coordinated amplification, fating and differentiation.

**Figure 3:**
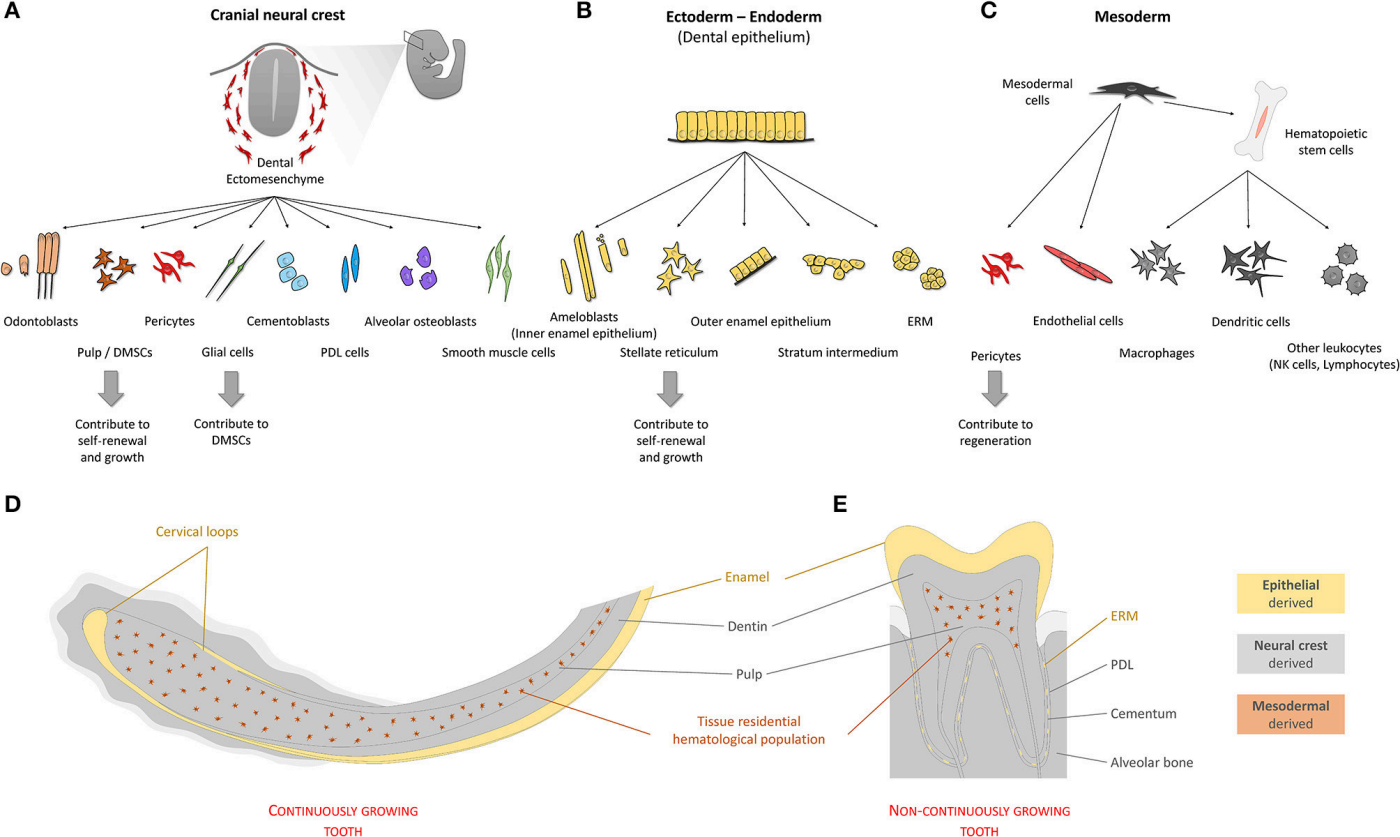
Origin of cells of the tooth. Teeth are organs composed by cells with different developmental origins. Quantitatively, most tooth tissue is of neural crest origin **(A)**. Cells emerging from the neural crest give rise to both pulp and extra-pulpal tissue. Dental epithelium can have ectodermal and/or endodermal origin. In the adult non-continuously growing tooth, dental epithelium-derived cells are absent, leaving enamel matrix that covers the tooth crown behind **(B)**. Continuously growing teeth maintain their SC niches and have all cell types mentioned above. In addition to the two main components, ectomesenchyme and epithelium, blood vessel-related tissue of mesodermal origin form fundamental parts of dental pulp tissue **(C)**. **(D,E)** Schematically depicted representation of tissues of different origins in continuously or non-continuously growing teeth. (PDL—periodontal ligament; SC's—stem cells; ERM—epithelial cell rests of Malassez; DMSCs—dental mesenchymal stem cells).

To summarize, manipulation of cell type composition, including residential progenitors and stem populations, appears as a cost-effective and highly flexible evolutionary strategy that can provide for any type of tooth renewal (Figure 3).

**Key papers:**

- Qu et al. (2015).
- Fraser et al. (2010).
- Soukup et al. (2008).
- Jernvall and Thesleff (2012).

## Author contributions

JK, IA, and KF: Framing the concept and structure, manuscript writing, approval of final manuscript. JK: Figures design. IA and KF: Editing and finalizing.

### Conflict of interest statement

The authors declare that the research was conducted in the absence of any commercial or financial relationships that could be construed as a potential conflict of interest. The reviewer PP and handling Editor declared their shared affiliation, and the handling Editor states that the process nevertheless met the standards of a fair and objective review.
